# Imported Cutaneous Melioidosis in Traveler, Belgium

**DOI:** 10.3201/eid1306.061460

**Published:** 2007-06

**Authors:** Khaled Ezzedine, Michel Heenen, Denis Malvy

**Affiliations:** * Hôpital Erasme, Brussels, Belgium; † University Hospital Center, Bordeaux, France

**Keywords:** Melioidosis, imported disease, inoculation lesion, Burkholderia pseudomallei, Belgium, letter

**To the Editor:** In some tropical areas, melioidosis, a disease caused by infection with *Burkholderia pseudomallei*, results in sepsis (*1*). This disease affects mostly adults with an underlying predisposing condition (*2*). With the increase in international travel, melioidosis has been identified in patients returning from disease-endemic areas (*3*). We report a case of a travel-associated cutaneous melioidosis without any systemic involvement.

A 90-year-old woman came to the Hôpital Erasme in Brussels with a nonhealing erythematous and ulcerated cutaneous lesion on the side of her left elbow. The lesion was a papule that gradually increased in size. The patient had diabetes mellitus but was otherwise healthy when she had traveled to Bangladesh 8 weeks earlier. She stayed 3 weeks in a village in the northwestern area of Rangpur District during the rainy season. She reported multiple insect and mosquito bites that evolved into intensely pruritic papules. This led to uncontrolled scratching and repeated washing of bite lesions with untreated well water.

Two weeks after her return, the lesion developed; it increased steadily in size, despite application of topical antimicrobial ointment. Three weeks later, after three visits to a physician, she was admitted to our institution. She did not report any fever, rigors, sweating, malaise, weight loss, or respiratory symptoms. Skin examination showed an irregular (3.0 cm × 4.0 cm), erythematous, fluctuant, tender, painful plaque (Appendix Figure, panel A). She did not have palpable regional lymph nodes. Results of a physical examination and laboratory investigations were normal. Five blood cultures at different times failed to isolate any microorganism.

A skin biopsy specimen from the plaque showed an inflammatory granulomatous reaction. Gram staining of biopsy specimens showed scanty lymphocytes, no polymorphonuclear leukocytes, and no microorganisms. Specimens were ground and placed on Columbia agar containing 5% horse blood and Schaedler enrichment broth and incubated aerobically for 3 days and on Schaedler agar containing 5% horse blood and incubated anaerobically for 10 days. After 72 hours, Schaedler broth showed a few colonies of an aerobic gram-negative bacillus that was identified as *B*. *pseudomallei* on the basis of typical biochemical characteristics. The strain was mobile at 37°C; grew at 42°C, oxidized but did not ferment glucose; produced cytochrome oxidase, arginine dihydrolase, and gelatinase; and was resistant to 300 IU polymyxin B 300 (DiaTabs; Rosco, Taastrup, Denmark). The isolate had a negative reaction for metabolism of arabinose.

Antimicrobial drug testing showed susceptibility to temocillin, amoxicillin-clavulanic acid (MIC 2 mg/L), piperacillin-tazobactam, ceftazidime, cefepime, meropenem (MIC 0.75 mg/L), doxycycline, and cotrimoxazole, and resistance to cefazolin, cefoxitin, ampicillin, gentamicin, ciprofloxacine, and amikacin. Results of tests for systemic involvement, as well as sputum and urine cultures, were negative. The patient was discharged and received oral doxycycline, 100 mg twice a day, and amoxicillin/clavulanic acid, 875 mg twice a day, for 32 weeks. The lesion dramatically improved 8 weeks after treatment was started (Appendix Figure, panel B) and had disappeared by 20 weeks after treatment was started (Appendix Figure, panel C). At 24 months after the diagnosis, no relapse had occurred.

Our patient with imported melioidosis had an unusual clinical course. She had never been febrile and had an uncomplicated localized skin infection skin despite her predisposing diabetes. A similar course has been reported in 2 tourists from Finland after the tsunami in Thailand in December 2004 (*4*), but most imported cases have pulmonary or systemic involvement associated with a severe prognosis (*5*,*6*).

The mode of acquisition in our patient herein was probably by an insect bite, contaminated water, or direct contact with wet soil during the rainy season. This mode of acquisition reinforces the hypothesis of a predominant role of percutaneous *B*. *pseudomallei* infection (*7*). Although the lesion healed, the patient was advised to have lifelong follow-up because relapses have been observed several years after infection.

Imported melioidosis is no longer a rare disease. With the increase in international travel and adventure tourism to disease-endemic regions, melioidosis is more likely to develop among travelers, even in those with short-term exposure. Recent reports suggest that melioidosis is probably widespread but poorly recognized throughout Bangladesh (*5*). Clinicians who treat patients returning from disease-endemic tropical areas, including the Indian subcontinent, should consider the disease in the differential diagnosis of febrile illnesses and isolated skin ulcers. Diagnosis is based on isolation of *B*. *pseudomallei* from blood, sputum, or biopsy specimens from lesions. Microbiologists should also be aware of the characteristics of the agent, and cultures should be handled under laboratory biosafety level 3 containment. Moreover, *B*. *pseudomallei* is a potential bioterrorism agent (*8*). Assessment of geographic and seasonal exposure is needed for identifiying this polymorphic exotic disease. Furthermore, travel advertisements to disease-endemic countries should include prophylactic measures to avoid contact with wet soils and contaminated water.

## Supplementary Material

Appendix Figure A) Irregular, erythematous, painful ulcerated plaque of the external side of the left elbow of the patient before treatment. B) Eight weeks after beginning treatment. C) Twenty weeks after beginning treatment.
