# Supplementary figures and images for: Imported Cutaneous Melioidosis in Traveler, Belgium
Source: Emerg Infect Dis. 2007 Jun;13(6):946–7. doi: 10.3201/eid1306.061460 (PMC2792863; doi:10.3201/eid1306.061460)

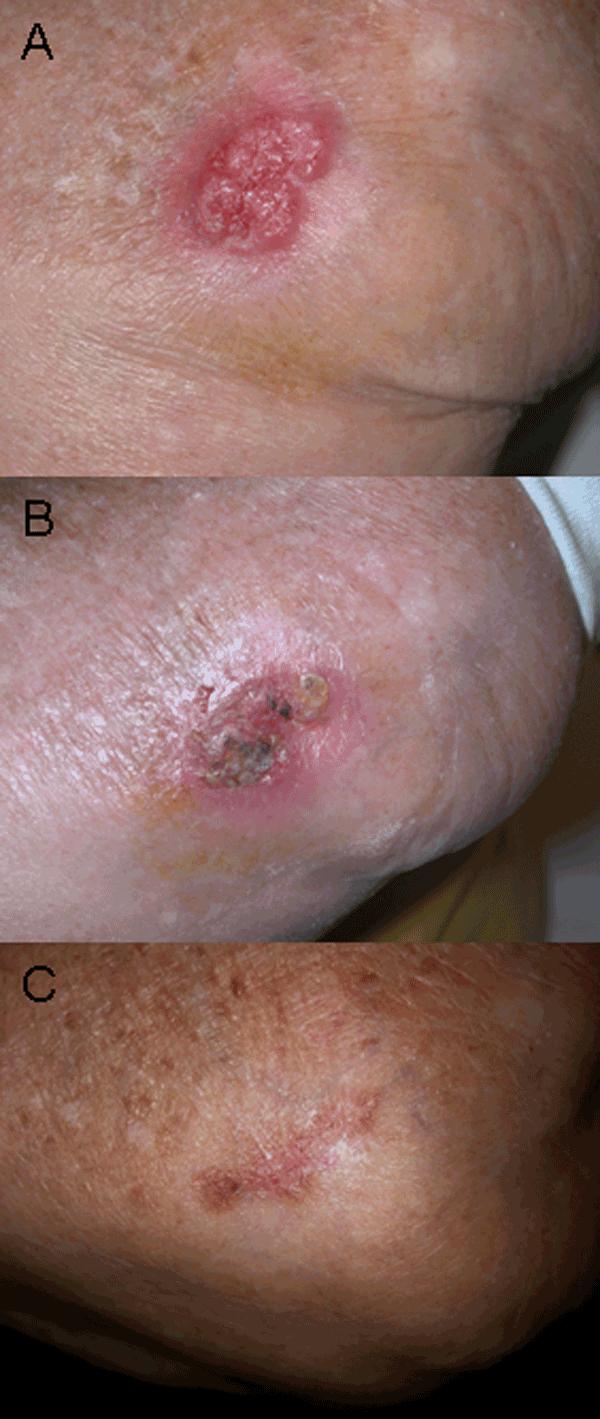

Supplement: Appendix Figure — A) Irregular, erythematous, painful ulcerated plaque of the external side of the left elbow of the patient before treatment. B) Eight weeks after beginning treatment. C) Twenty weeks after beginning treatment. [file 06-1460_appF-s1.gif]
